# Role of 99mTc-DTPA Captopril Renal Scintigraphy in the Diagnosis of Renal Fibromuscular Dysplasia: A Case Report

**DOI:** 10.7759/cureus.105735

**Published:** 2026-03-23

**Authors:** Gabriel Infante, David Gutierrez Albenda, Ana María Gutiérrez, Mariana Parra, Ian Taylor

**Affiliations:** 1 General Practice, University of Costa Rica, San José, CRI; 2 Cyclotron-PET/CT Laboratory, University of Costa Rica, San José, CRI; 3 General Medicine, University of Costa Rica, San José, CRI; 4 Medical School, University of Costa Rica, San José, CRI

**Keywords:** 99mtc-dtpa scintigraphy, captopril, fibromuscular dysplasia, glomerular filtration rate, renal artery stenosis, renovascular hypertension, secondary hypertension

## Abstract

Fibromuscular dysplasia (FMD) represents the most common non-inflammatory and non-atherosclerotic cause of renal artery stenosis (RAS), frequently manifesting as resistant renovascular hypertension (RVH). While anatomical imaging can identify structural abnormalities such as the classic "string-of-beads" pattern, 99mTc-DTPA captopril renal scintigraphy provides essential physiological data by demonstrating a measurable drop in the glomerular filtration rate (GFR) when the compensatory renin-angiotensin-aldosterone system is disrupted. This case report details a 33-year-old male with severe hypertension (190/140 mmHg) and secondary hyperaldosteronism whose diagnosis was supported by baseline and post-captopril scintigraphy, showing a reduction in differential renal function. Subsequent renal angiography confirmed a 50% stenotic lesion. Balloon angioplasty was complicated by distal dissection, necessitating stent placement, and the patient subsequently achieved symptom resolution with discontinuation of antihypertensive therapy. With a reported sensitivity of 83-93% and a specificity of 90%, 99mTc-DTPA scintigraphy serves as a highly effective, non-invasive diagnostic tool that captures the functional significance of renal lesions, potentially avoiding more invasive procedures while guiding early intervention to prevent permanent kidney injury.

## Introduction

Fibromuscular dysplasia (FMD) is the most common non-inflammatory and non-atherosclerotic cause of renal artery stenosis (RAS). FMD leads to various vascular abnormalities, including dissection, aneurysm, or stenosis [1]. The etiology of FMD remains unknown, but it has been associated with genetic and environmental factors, such as smoking, mechanical factors, stretching, and vasculotoxic medications, such as fluoroquinolones [2]. One study associated the presence of HLA-DRw6 histocompatibility antigen with the pathogenesis of the disease [3]. FMD is most common in young and middle-aged women; cases in males are less common, albeit frequently reported in the medical literature. In contrast to atherosclerotic renal artery disease, the primary cause of RAS, FMD is typically associated with resistant hypertension and seldom with a loss of renal function [1,4]. Clinically, FMD should be suspected in young patients presenting with severe or resistant hypertension, particularly when laboratory findings suggest activation of the renin-angiotensin-aldosterone system (RAAS) or when imaging reveals renal artery abnormalities in the absence of atherosclerotic risk factors [3,5].

The radiographic presentation that is suggestive of FMD as the cause of RAS consists of a “string-of-beads” pattern on the renal artery involved and the International FMD Consensus defines that ≥1 multifocal or focal arterial lesion must be present; however, the absence of these lesions is not enough to rule out the diagnosis, even if aneurysm, dissection or tortuosity are present [1,2]. There are many imaging modalities to establish a diagnosis of FMD, such as Doppler ultrasound (DU), magnetic resonance angiography (MRA), and computed tomography angiography (CTA) [5]. The gold standard in this regard is catheter angiography (CA), and the accuracy can be improved with the use of concomitant intravascular ultrasound (IVUS) [3,6]. Nevertheless, none of these studies is able to describe the physiological impairment of renal function associated with the anatomical aberrations [7,8].

Renal scintigraphy, a functional nuclear medicine scan, is used to quantify the differential function of each kidney and observe if there is any obstruction. Specifically, Technetium-99m diethylenetriaminepentaacetic acid (99mTc-DTPA) with captopril has been proven to be an effective study to diagnose RAS with a high sensitivity [7-9]. The underlying principle of 99mTc-DTPA scintigraphy is to perform an initial baseline study, followed by a second scan after administering captopril. Administering an angiotensin-converting enzyme (ACE) inhibitor like captopril disrupts the compensatory RAAS. In a stenotic kidney, GFR is maintained by angiotensin II-mediated vasoconstriction of the efferent arteriole; captopril removes this compensation, leading to a measurable drop in GFR [9]. A relative change in 99mTc-DTPA uptake greater than 10% or a reduction of more than 10% in GFR after captopril administration is highly suggestive of FMD [9]. This study can help to avoid doing a diagnostic angiography or even to show the improvement after angioplasty or reconstructive surgery [8,9]. In this case report, we present a case of FMD of the right renal artery causing RVH diagnosed by 99mTc-DTPA scintigraphy with captopril. A brief review of the medical literature that has been published regarding the use of 99mTc-DTPA scintigraphy for these purposes.

## Case presentation

A 33-year-old male presented with recent-onset hypertension. His medical history was unremarkable, with no history of tobacco or alcohol use. Both parents had been diagnosed with hypertension before the age of 50. He reported a four-month history of episodic diarrhea, nausea, vomiting, and headaches. During one episode, his blood pressure was 190/140 mmHg (reference: <120/80 mmHg). A 24-hour ambulatory blood pressure monitoring study showed a mean blood pressure of 140/92 mmHg (reference: <130/80 mmHg), with persistently elevated values during sleep.

The patient was initially managed with losartan/hydrochlorothiazide, which was increased to twice daily. During workup for secondary hypertension, a routine laboratory examination, shown in Table 1, was made. Secondary hyperaldosteronism was suggested by an elevated renin level, a normal-to-high aldosterone level, and a low aldosterone-to-renin ratio of 1.3 (calculated by dividing aldosterone concentration by plasma renin activity). Tests to exclude other secondary causes of hypertension were negative, including 24-hour urinary 5-HIAA, 24-hour urinary free cortisol, and plasma metanephrines.

**Table 1:** Routine laboratory examination

	Patient’s result	Reference
Renin (µU/mL)	243.8	≤39.9
Plasma renin activity (ng/mL/h)	20.3	0.2-1.6
Aldosterone (ng/dL)	27.6	≤39.2
Aldosterone-to-renin ratio	1.3	<20
Metanephrine (nmol/L)	0.3	<0.5
Normetanephine (nmol/L)	0.4	<0.9
24-hour urinary 5-hidroxiindolacetic acid (mg/24h)	2.2	2-6
24-hour urinary free cortisol (µg/24h)	32.8	≤60.0
Creatinine (mg/dL)	1.1	0.1-1.2

**Figure 1:**
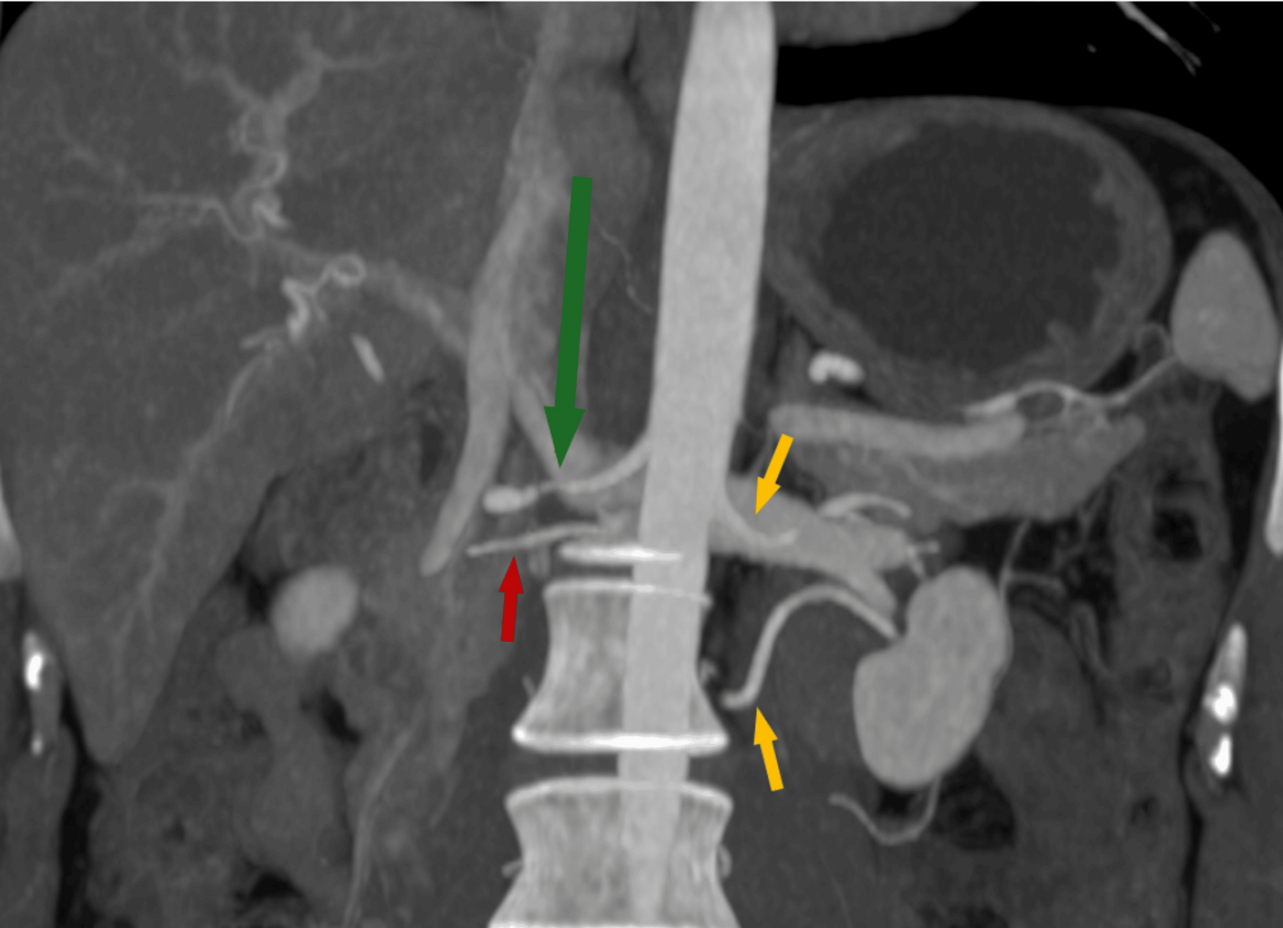
Coronal maximum intensity projection (MIP) reconstruction The right main renal artery (green arrow) shows moderate focal stenoses starting 1.7 cm from its aortic origin, remaining patent. A right accessory renal artery (red arrow) arises 1.1 cm inferior to the main artery. Two left renal arteries (yellow arrows) have preserved caliber and course.

An abdominal CT scan (Figure 1) showed parietal irregularities and sequential moderate stenosis of the right renal artery, associated with delayed contrast concentration in the upper third of the right kidney, findings highly compatible with a diagnosis of FMD.

**Figure 2:**
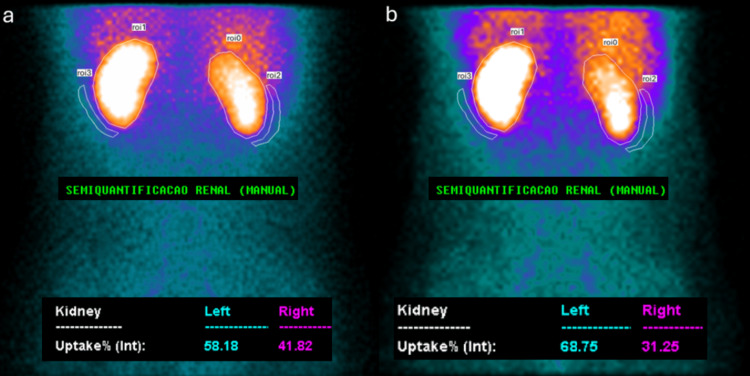
(a) Baseline 99mTc-DTPA renal scintigraphy demonstrating a semi-quantitative differential renal function of 42% for the right kidney and 58% for the left kidney. (b) Captopril-enhanced 99mTc-DTPA renal scintigraphy showing a semi-quantitative differential renal function of 31% for the right kidney and 69% for the left kidney.

A baseline dynamic renal scintigraphy, shown in Figure 2a, was performed, demonstrating preserved tracer uptake and excretion in the left kidney and mildly reduced perfusion on the right. The semi-quantitative differential renal function at baseline was calculated at 42% for the right kidney and 58% for the left kidney, without significant tracer retention or delayed time to peak activity.

A captopril-enhanced 99mTc-DTPA renal scintigraphy, shown in Figure 2b, was subsequently obtained. In comparison with the baseline study, the post-captopril scan demonstrated asymmetric arterial renal perfusion, moderately reduced on the right and preserved on the left. Semi-quantitative differential renal function declined to 31% in the right kidney, with a corresponding increase to 69% in the left kidney. This represents an absolute decline of 11% in differential renal function, exceeding the commonly accepted diagnostic threshold of 10% following captopril administration and supporting the presence of hemodynamically significant RAS.

**Figure 3:**
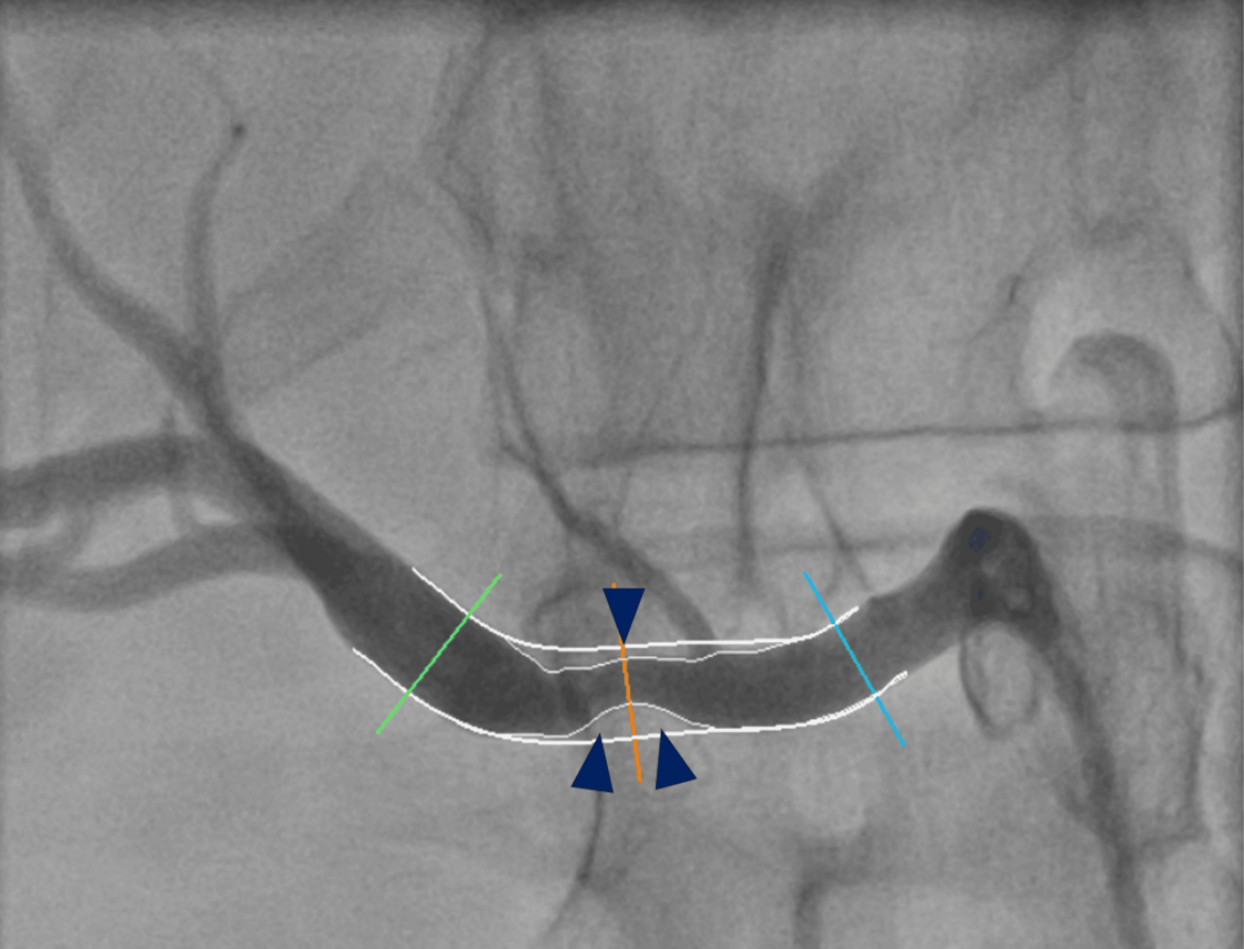
Right renal arteriography. The right superior renal artery demonstrates an approximately 50% stenotic lesion in its mid-third (blue arrowheads).

Renal angiography (Figure 3), performed with the intent of proceeding to balloon angioplasty, confirmed an approximately 50% stenotic lesion in the right renal artery. The intervention was complicated by a distal arterial dissection, necessitating bail-out stent placement to maintain vessel patency. After successful revascularization, the patient experienced complete symptom resolution and was able to discontinue all antihypertensive medications. At the final follow-up, he remained asymptomatic with sustained optimal blood pressure control.

## Discussion

Current epidemiology and classification of RAS

RAS is one of the most common causes of secondary hypertension, most commonly presenting as RVH [7,9]. RVH represents 1-5% of all the cases of hypertension in the general population [5,10], and in selected groups, the number of patients could be higher [6]. The principal causes of RVH include atherosclerosis, which causes, on average, 90% of RAS cases and affects prior groups with cardiovascular risk, such as patients who smoke or have diabetes [4,10]. FMD is the second most common cause, with an incidence of between 10% and 30% of cases, and is most frequent in children or young patients [3,5]. Less than 5% of the cases are made because of rare conditions such as type 1 neurofibromatosis, Williams-Beuren syndrome, Alagille syndrome, Takayasu arteritis, and segmentary arterial mediolysis [4].

FMD was considered an infrequent finding, but recent studies in kidney donors showed that the prevalence of FMD was between 2.3% and 6.6% [1,6]. It affects women more often than men, with a percentage of women affected in 82-95% of all cases and an average age of diagnosis of 43-53 years old. This disease has a systemic behavior, where it not only affects renal arteries in 66-91% of cases, but also cerebral arteries in 25-80% of cases, and in lower percentages, mesenteric and lower limbs arteries [2].

The classification of RAS can be performed by the etiology, which can be divided into atherosclerotic and non-atherosclerotic lesions. The first group often affects the ostium and the proximal segment of the renal artery. Meanwhile, the non-atherosclerotic group, which includes FMD, is used for diseases that affect the distal and medial segments of the renal artery [3,5,6].

Recognizing FMD as the second most common and highly probable cause of RAS is essential, particularly in cases of renovascular hypertension (RVH) that respond poorly to multiple antihypertensive agents. FMD represents a potentially treatable etiology; early diagnosis is crucial to prevent irreversible renal damage in the affected kidney and to reduce the risk of cardiovascular complications [7].

Current evidence regarding the use of 99mTc-DTPA scintigraphy with captopril in the diagnosis of fibromuscular dysplasia

99mTc-DTPA scintigraphy with captopril is a valuable tool for diagnosing RVH caused by FMD [5,8]. While other study methods, such as DU, MRA, and CTA, demonstrate only anatomical abnormalities, scintigraphy provides functional evidence of impaired renal perfusion and filtration. Moreover, in some cases, it can help to avoid invasive procedures such as percutaneous intervention for the diagnosis [7,9]. The sensitivity of 83-93% and specificity of 90% makes this study trustworthy for the diagnosis [8,9].

Three standard scintigraphic criteria suggestive of hemodynamically significant RAS include [7,9] 1) a relative reduction of uptake >10% in the affected kidney after the administration of the captopril, 2) a reduction of >10% in the calculated GFR in the ipsilateral kidney, and 3) retention of the 99mTc-DTPA on the kidney parenchyma and a prolonged time to reach maximum activity (Tmax).

It is also important to know the limitations of this study method in the context of FMD. In multifocal FMD, renal blood flow and renin secretion may be relatively normal. The diagnostic yield of scintigraphy depends on hemodynamic significance. Consequently, mild stenosis may result in false negatives [1,8]. Furthermore, bilateral FMD, azotemia, or a solitary functioning kidney can diminish the scan’s sensitivity and specificity; also, the presence of azotemia and FMD in a mono-renal patient could also reduce these parameters [7,10]. While 99mTc-DTPA is commonly used for the evaluation of glomerular filtration rate (GFR) because it is filtered exclusively by the glomerulus, other tracers may be preferable in specific situations. 99mTc-MAG3 is often preferred in patients with impaired renal function because it has a significantly higher renal extraction fraction due to active tubular secretion. This characteristic allows better image quality and more reliable functional assessment in patients with reduced glomerular filtration, whereas DTPA uptake may be limited when renal function is significantly decreased [8].

## Conclusions

This case illustrates the clinical utility of 99mTc-DTPA captopril renal scintigraphy as a non-invasive tool in the evaluation of RVH caused by FMD. 99mTc-DTPA scintigraphy with captopril is a highly effective and non-invasive tool for diagnosing RVH caused by FMD. It offers a high sensitivity of 83-93% and a specificity of 90%. 

Unlike anatomical imaging modalities such as CTA or MRA, which only show structural changes, scintigraphy demonstrates the actual pathophysiological impact of the stenosis on renal function. This study can help clinicians avoid invasive diagnostic procedures and provide a clear way to monitor improvements after interventions such as angioplasty or reconstructive surgery. In addition, scintigraphy allows quantification of the relative contribution of each kidney to the total GFR. Successful treatment of FMD-related stenosis should result in an increase in the relative function of the previously stenotic kidney. Currently, there is limited evidence regarding the optimal timing and frequency of post-treatment scintigraphic follow-up. Further studies are needed to establish its diagnostic performance in the post-intervention setting and to compare its utility with the current standard practice of annual DU surveillance.

Identifying FMD early is crucial because it is a treatable cause of hypertension; timely diagnosis prevents established kidney injury and serious cardiovascular complications. Although FMD predominantly affects middle-aged women, this case highlights the importance of considering the diagnosis even in younger male patients presenting with severe or resistant hypertension. It is important to note that diagnostic accuracy may be limited by certain factors, including mild stenosis (which may cause false negatives), bilateral FMD, or pre-existing renal impairment such as azotemia.
